# Visualizing spatiotemporal dynamics of apoptosis after G1 arrest by human T cell leukemia virus type 1 Tax and insights into gene expression changes using microarray-based gene expression analysis

**DOI:** 10.1186/1471-2164-13-275

**Published:** 2012-06-22

**Authors:** Mariluz Arainga, Hironobu Murakami, Yoko Aida

**Affiliations:** 1 Viral Infectious Diseases Unit, RIKEN, 2-1 Hirosawa, Wako, Saitama, 351-0198, Japan; 2 Department of Medical Genome Sciences, Graduate School of Frontier Science, Laboratory of Viral Infectious Diseases, The University of Tokyo, 2-1 Hirosawa, Wako, Saitama, 351-0198, Japan; 3 Japan Foundation for AIDS Prevention, Chiyoda-ku, Tokyo, Japan

## Abstract

**Background:**

Human T cell leukemia virus type 1 (HTLV-1) Tax is a potent activator of viral and cellular gene expression that interacts with a number of cellular proteins. Many reports show that Tax is capable of regulating cell cycle progression and apoptosis both positively and negatively. However, it still remains to understand why the Tax oncoprotein induces cell cycle arrest and apoptosis, or whether Tax-induced apoptosis is dependent upon its ability to induce G_1_ arrest. The present study used time-lapse imaging to explore the spatiotemporal patterns of cell cycle dynamics in Tax-expressing HeLa cells containing the fluorescent ubiquitination-based cell cycle indicator, Fucci2. A large-scale host cell gene profiling approach was also used to identify the genes involved in Tax-mediated cell signaling events related to cellular proliferation and apoptosis.

**Results:**

Tax-expressing apoptotic cells showed a rounded morphology and detached from the culture dish after cell cycle arrest at the G_1_ phase. Thus, it appears that Tax induces apoptosis through pathways identical to those involved in G_1_ arrest. To elucidate the mechanism(s) by which Tax induces cell cycle arrest and apoptosis, regulation of host cellular genes by Tax was analyzed using a microarray containing approximately 18,400 human mRNA transcripts. Seventeen genes related to cell cycle regulation were identified as being up or downregulated > 2.0-fold in Tax-expressing cells. Several genes, including SMAD3, JUN, GADD45B, DUSP1 and IL8, were involved in cellular proliferation, responses to cellular stress and DNA damage, or inflammation and immune responses. Additionally, 23 pro- and anti-apoptotic genes were deregulated by Tax, including TNFAIP3, TNFRS9, BIRC3 and IL6. Furthermore, the kinetics of IL8, SMAD3, CDKN1A, GADD45A, GADD45B and IL6 expression were altered following the induction of Tax, and correlated closely with the morphological changes observed by time-lapse imaging.

**Conclusions:**

Taken together, the results of this study permit a greater understanding of the biological events affected by HTLV-1 Tax, particularly the regulation of cellular proliferation and apoptosis. Importantly, this study is the first to demonstrate the dynamics of morphological changes during Tax-induced apoptosis after cell cycle arrest at the G_1_ phase.

## Background

Human T cell leukemia virus type 1 (HTLV-1) causes adult T cell leukemia (ATL), a severe and fatal lymphoproliferative disease of helper T cells [1], and a separate neurodegenerative disease called tropical spastic paraparesis/HTLV-1-associated myelopathy (TSP/HAM) [2]. HTLV-1 encodes a 40 kDa regulatory protein, Tax, which is necessary and sufficient for cellular transformation and is, therefore, considered to be the viral oncoprotein. Tax is a potent activator of both viral and cellular gene expression, and the oncogenic potential of Tax is thought to depend on its ability to alter the expression of cellular genes involved in cell growth and proliferation, and its direct interactions with cell cycle regulators [3,4]. Tax-mediated transcriptional activation of cellular gene expression requires direct contact with components of the cyclic AMP-response element binding protein (CREB), nuclear factor-κB (NF-κB), and the serum response factor (SRF) signaling pathways [5]. Moreover, Tax is thought to be involved in other cellular processes including DNA repair, cell cycle progression, and apoptosis [6,7].

Tax stimulates cell growth via cell cycle dysregulation [3,4,7]. A major mitogenic activity of Tax is stimulation of the G_1_-to-S-phase transition [8-12], and several different mechanisms have been proposed to explain the dysregulation of the G_1_ phase and the accelerated progression into S phase. In mammalian cells, G_1_ progression is controlled by the sequential activation of the cyclin-dependent kinases (Cdks) Cdk4, Cdk6, and Cdk2. Activation of these Cdks by Tax leads to hyperphosphorylation of Retinoblastoma (Rb) and the liberation of E2F, which is essential for cell cycle progression [12,13]. Tax interacts with cyclins D1, D2, and D3, but not with Cdk1 or Cdk2 [11,14-16]. By binding to cyclins, Tax stabilizes the cyclin D/Cdk complex, thereby enhancing its kinase activity and leading to the hyperphosphorylation of Rb. Moreover, Tax activates the transcription of cyclin D1 and D2 [17,18] by deregulating the NF-κB pathway [18,19]. By contrast, there is evidence that Tax induces cell cycle arrest at the G_1_ phase [20]. HTLV-1 infection and Tax expression in human cells have been observed to induce cell cycle arrest at the G_1_ phase by inducing p27/kip1 and p21/waf1 [20], and the sharp rise in p27 induced by Tax is often associated with premature activation of the anaphase-promoting complex (APC) [21]. Indeed, cells infected with HTLV-1 expressing wild-type Tax arrest at the G_1_/S boundary when subjected to cellular stress [22,23].

Interestingly, Tax induces apoptosis in a variety of systems [24-26], consistent with its ability to inhibit DNA repair. Indeed, HTLV-1-infected cells undergo increased apoptosis upon cellular stress [22-28]; however, other reports show that Tax inhibits apoptosis [29-31], supporting its role as a transforming protein and an inducer of T cell proliferation. Therefore, it seems likely that Tax is capable of stimulating both pro- and anti-apoptotic pathways.

Tax regulates cell cycle progression and apoptosis both positively and negatively; however, the molecular mechanism(s) underlying the regulation of these processes by Tax remain obscure. In this study, we examined the regulation of cell cycle progression and apoptosis by Tax and demonstrated the following: (i) a high level of transient Tax expression arrests the cell cycle at the G_1_ phase and induces apoptosis in HeLa cells; (ii) based on a microarray containing approximately 18,400 human mRNA transcripts, genes related to cell cycle progression and apoptosis were deregulated by Tax in HeLa cells; (iii) time-lapse imaging of a fluorescent ubiquitination-based cell cycle indicator (Fucci2) in HeLa cells allows for dual-color imaging and can be used to distinguish between live cells in the G_1_ and S/G_2_/M phases. Using this system for the *in vivo* analysis of the spatial and temporal patterns of cell cycle dynamics [32,33], we demonstrated that Tax-expressing cells arrest in the G_1_ phase of the cell cycle and proceeded to apoptosis; and (iv) we found that Tax-induced changes in the expression of genes related to cell cycle regulation and apoptosis correlated well with the morphological changes observed in the cells.

## Results

### Tax induces cell cycle arrest and apoptosis in transfected HeLa cells

**Figure 1:**
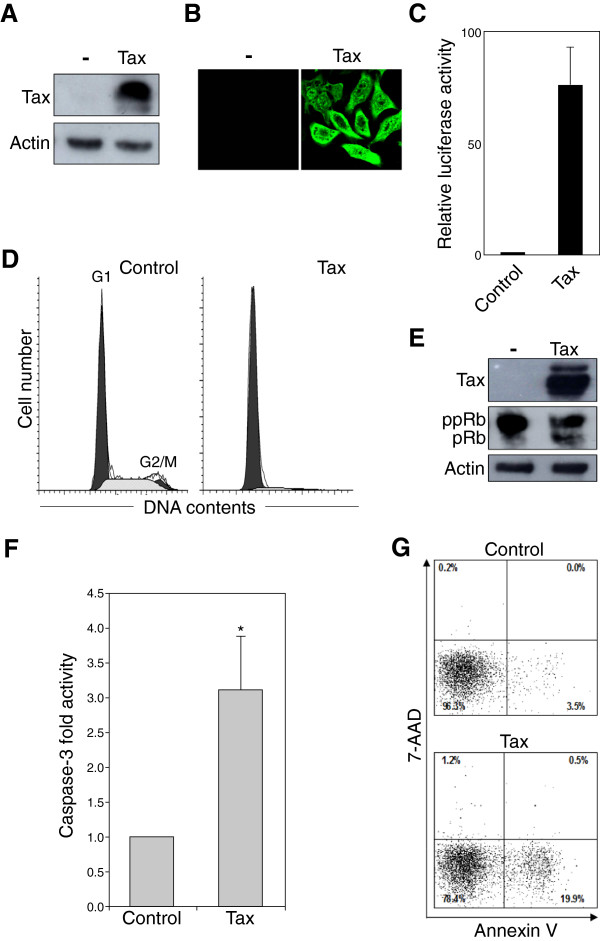
**Tax induces G**_**1**_**cell cycle arrest and apoptosis.** HeLa cells were transiently transfected with a pCAGGS-Tax Flag-tagged vector or the control pCAGGS vector (**A, B** and **E**) together with either the reporter plasmid pGV-HL21 (HTLV-1 enhancer) and the reference plasmid pRL-SV40 (**C**), or the GFP expression vector pEGFP-NI (**D** and **G**) or the pSV-β-galactosidase vector (**F**). (A) At 24 h post-transfection, cells were lysed and subjected to immunoblot analysis with an anti-Flag MAb and an anti-actin MAb (as a control). (**B**) At 24 h post-transfection, cells were fixed, permeabilized, and immunostained with an anti-Flag MAb followed by an Alexa 488-conjugated anti-mouse IgG antibody. Cells were analyzed by confocal laser scanning microscopy (Olympus FV1000). (**C**) At 48 h after transfection, cells were recovered and the activities of firefly and *Renilla* luciferases were measured in lysates. For each sample, the firefly luciferase activity (pGV-HL21) was normalized by reference to *Renilla* luciferase activity (pRL-SV40). (**D**) At 48 h post-transfection, cells were fixed and stained with propidium iodide for the analysis of DNA content. GFP-positive cells were analyzed by flow cytometry using Cell Quest for acquisition and ModFit LT. The peaks of the cells at G_1_ and G_2_/M phase are indicated. (**E**) At 48 h post-transfection, cells were collected, lysed, and analyzed for phosphorylation of Rb by immunoblotting with an anti-Rb MAb using an anti-actin MAb as a control. ppRb, hyperphosphorylated forms of Rb; pRb, hypo- and unphosphorylated forms of Rb. (F) At 48 h post-transfection, cells were collected, lysed, β-galactosidase activity was measured. Caspase-3 activity was measured in the cell lysates with an equal amount of β-galactosidase activity. Each of the columns and its associated error bar represent the mean ± standard deviation (SD) of results from four different experiments. The asterisk (*) represents a *p*-value of < 0.01. (G) At 48 h post-transfection, cells were stained with PE-Annexin V and 7-AAD to identify apoptotic cells. GFP was used as a reporter to discriminate between transfected and untransfected cells. The percentage of Annexin V-positive and 7-AAD-negative cells relative to GFP-positive cells indicates the level of apoptosis.

To examine whether Tax induces cell cycle arrest at the G_1_ phase and promotes apoptosis in HeLa cells, chimeric Tax carrying a Flag tag at the carboxyl terminus was transfected into HeLa cells. At 24 h post transfection, the expression of Tax protein was assessed by immunoblot analysis of cell extracts using the monoclonal antibody (MAb) M2, which recognizes the Flag tag (Figure 1A). A single band with an apparent molecular mass consistent with the predicted sequences was observed. As shown in Figure 1B, Tax was detected in both the nucleus and cytoplasm of transfected HeLa cells. This result correlates well with previous studies indicating that Tax is able to shuttle between the nucleus and the cytoplasm but predominantly localizes in the nucleus [34]. As shown in Figure 1C, Tax showed considerable transactivation activity toward the HTLV-1 enhancer, indicating that chimeric Tax with a C-terminal Flag tag was fully functional.

Next, the cell cycle distribution of Tax-expressing HeLa cells was analyzed. Cells were stained with propidium iodide (PI) and analyzed by flow cytometry 48 h after co-transfection with the Tax expression vector or the control vector and a green fluorescence protein (GFP) expression vector, pEGFP-N1, which served as a marker plasmid. The histograms show representative data from one of three independent experiments. As shown in Figure 1D, flow cytometry analysis revealed that there was a marked increase in the percentage of cells in the G_1_ phase in cells transfected with Tax (approximately 92% ± 4.5%) compared with cells transfected with the control vector (approximately 58% ± 4.3%), strongly indicating that G_1_ cell cycle arrest was induced in Tax-expressing cells (p < 0.001). To confirm this result, total cell extracts were collected 48 h post-transfection and the phosphorylation status of Rb was determined by immunoblotting with an anti-Rb MAb, which detects all forms of Rb. The phosphorylation status of Rb serves as a marker of cells in the G_0_/G_1_ phase of the cell cycle, since Rb is progressively phosphorylated throughout the G_1_ phase and is hyperphosphorylated upon transition into the S phase [35]. As shown in Figure 1E, hyperphosphorylated form (ppRb) migrated more slowly than the hypo- and unphosphorylated forms (pRb). The majority of Rb was hyperphosphorylated (upper major band) in cells transfected with the control vector; however, a decrease in the level of hyperphosphorylated form (ppRb) and an increase in the levels of hypo- and/or unphosphorylated form (pRb) were observed in extracts prepared from Tax-expressing cells. These results confirmed that Tax prevents hyperphosphorylation of Rb and blocks cell cycle progression at the G_1_ phase.

To analyze whether Tax induced apoptosis, HeLa cells were transfected with a Tax expression vector or a control vector, and the activity of caspase-3, which plays an essential role in apoptosis, was measured. Caspase-3 activity was significantly higher in Tax-expressing cells than in control cells (Figure 1F; p < 0.01). Next, the apoptotic activity of Tax was further quantified using flow cytometry by co-staining transfected cells with phycoerythrin (PE)-Annexin V and 7-amino-actinomycin D (7-AAD) (Figure 1G). A prominent event in early apoptosis is the exposure of phosphatidylserine (PS) on the outer leaflet of the cell membrane. Cell surface-exposed PS is specifically detected by PE-Annexin V, and during the late stages of apoptosis or necrosis, cell membrane integrity is lost, allowing entry of the DNA-binding dye 7-AAD. The population of Annexin V-positive and 7-AAD-negative apoptotic cells was much higher in Tax-expressing cells (19.9%) than in cells transfected with the control vector (3.5%). Because the same trends were observed for caspase-3 activity (Figure 1F) and apoptotic activity (Figure 1G), it was concluded that Tax induces apoptosis in HeLa cells.

### Large-scale expression profiling of cellular genes after transfection with tax

To analyze the mechanism(s) underlying the regulation of cell cycle progression and apoptosis by Tax, total RNA was isolated from HeLa cells transfected with Tax or a control vector, and each RNA sample was subjected to microarray analysis (GEO accession number GSE34750). Data sets were analyzed using GeneSpring GX 11.0 software for gene expression, clustering, gene ontology, and significant signaling pathways. Using microarrays containing approximately 18,400 mRNA transcripts, 342 genes were identified (269 upregulated and 73 downregulated) that showed statistically significant levels of differential regulation by Tax (p < 0.05) (Tables 1 and 2).

**Table 1:** Genes upregulated by Tax (fold change ≥ 2.0, p < 0.05)

**Gene symbol**	**Gene description**	**Gene ID**	**Fold change**
	***Transcription/Translation/RNA processing***		
FOXF1	forkhead box F1	2294	2.0
NFKB2	nuclear factor of kappa light polypeptide gene enhancer in B-cells 2 (p49/p100)	4791	2.0
NR6A1	nuclear receptor subfamily 6, group A, member 1	2649	2.0
CEBPD	CCAAT/enhancer binding protein (C/EBP), delta	1052	2.1
ETV5	ets variant 5	2119	2.1
FST	follistatin	10468	2.2
KLF6	Kruppel-like factor 6	1316	2.2
CEBPD	CCAAT/enhancer binding protein (C/EBP), delta	1052	2.3
EGR3	early growth response 3	1960	2.3
SAMD4A	sterile alpha motif domain containing 4A	23034	2.3
ELL2	elongation factor, RNA polymerase II, 2	22936	2.4
HIVEP2	human immunodeficiency virus type I enhancer binding protein 2	3097	2.4
MAFB	v-maf musculoaponeurotic fibrosarcoma oncogene homolog B (avian)	9935	2.4
FOSL2	FOS-like antigen 2	2355	2.5
ID2	inhibitor of DNA binding 2, dominant negative helix-loop-helix protein	3398	2.5
KLF2	Kruppel-like factor 2 (lung)	10365	2.5
RELB	v-rel reticuloendotheliosis viral oncogene homolog B	5971	2.5
MAFF	v-maf musculoaponeurotic fibrosarcoma oncogene homolog F (avian)	23764	2.8
LARP6	La ribonucleoprotein domain family, member 6	55323	3.1
REL	v-rel reticuloendotheliosis viral oncogene homolog (avian)	5966	3.1
FOSB	FBJ murine osteosarcoma viral oncogene homolog B	2354	3.2
HES1	hairy and enhancer of split 1 (Drosophila)	3280	3.2
SOD2	superoxide dismutase 2, mitochondrial	6648	3.4
ATF3	activating transcription factor 3	467	3.6
FOSL1	FOS-like antigen 1	8061	4.1
ZFP36	zinc finger protein 36, C3H type, homolog (mouse)	7538	4.2
ZNF331	zinc finger protein 331	55422	4.4
BACH2	BTB and CNC homology 1, basic leucine zipper transcription factor 2	60468	4.4
NFKBIE	nuclear factor of kappa light polypeptide gene enhancer in B-cells inhibitor, epsilon	4794	4.4
EGR1	early growth response 1	1958	6.2
FOS	FBJ murine osteosarcoma viral oncogene homolog	2353	8.4
NR4A2	nuclear receptor subfamily 4, group A, member 2	4929	10.9
	***Signal Transduction***		
EDN2	endothelin 2	1907	2.0
EPHA2	EPH receptor A2	1969	2.1
RIT1	Ras-like without CAAX 1	6016	2.1
SH2D3A	SH2 domain containing 3A	10045	2.1
KLRC1	killer cell lectin-like receptor subfamily C, member 1///killer cell lectin-like receptor subfamily C, member 2	3821	2.2
PSD4	pleckstrin and Sec7 domain containing 4	23550	2.5
ADM	adrenomedullin	133	2.7
GPRC5C	G-protein-coupled receptor, family C, group 5, member C	55890	2.7
BDKRB2	bradykinin receptor B2	624	2.8
GDF15	growth differentiation factor 15	9518	3.0
GPR87	G protein-coupled receptor 87	53836	3.4
RASA4	RAS p21 protein activator 4///RAS p21 protein activator 4 pseudogene	10156	8.8
GEM	GTP binding protein overexpressed in skeletal muscle	2669	14.1
GABBR1	gamma-aminobutyric acid (GABA) B receptor, 1///ubiquitin D	10537	24.5
RRAD	Ras-related associated with diabetes	6236	115.2
	***Inflammatory response/Immune response***		
KLRC1	killer cell lectin-like receptor subfamily C, member 1	3821	2.2
PTGS2	prostaglandin-endoperoxide synthase 2 (prostaglandin G/H synthase and cyclooxygenase)	5743	2.3
CCL22	chemokine (C-C motif) ligand 22	6367	3.0
IL6R	interleukin 6 receptor	3570	3.0
TNIP1	TNFAIP3 interacting protein 1	10318	3.2
EBI3	Epstein-Barr virus induced 3	10148	3.2
IL27RA	interleukin 27 receptor, alpha	9466	3.5
TRIM22	tripartite motif-containing 22	10346	3.8
IL32	interleukin 32	9235	3.8
TNFAIP6	tumor necrosis factor, alpha-induced protein 6	7130	4.0
GBP2	guanylate binding protein 2, interferon-inducible	2634	5.2
GBP2	guanylate binding protein 2, interferon-inducible	2634	5.4
CCL19	chemokine (C-C motif) ligand 19	6363	6.2
CXCL1	chemokine (C-X-C motif) ligand 1 (melanoma growth stimulating activity, alpha)	2919	11.7
CXCL11	chemokine (C-X-C motif) ligand 11	6373	13.1
CXCL3	chemokine (C-X-C motif) ligand 3	2921	19.1
CCL20	chemokine (C-C motif) ligand 20	6364	20.1
PTX3	pentraxin-related gene, rapidly induced by IL-1 beta	5806	81.1
CXCL2	chemokine (C-X-C motif) ligand 2	2920	87.0
	***Apoptosis regulation***		
ZMAT3	zinc finger, matrin type 3	64393	2.0
JMJD6	jumonji domain containing 6	23210	2.0
AEN	apoptosis enhancing nuclease	64782	2.1
ADORA2A	adenosine A2a receptor	135	2.2
CD70	CD70 molecule	970	2.2
FAS	Fas (TNF receptor superfamily, member 6)	355	2.3
BAG3	BCL2-associated athanogene 3	9531	2.4
BIK	BCL2-interacting killer (apoptosis-inducing)	638	2.4
BCL6	B-cell CLL/lymphoma 6	604	2.7
TNFRSF1B	tumor necrosis factor receptor superfamily, member 1B	7133	3.3
ZC3H12A	zinc finger CCCH-type containing 12A	80149	4.1
NFKBIA	nuclear factor of kappa light polypeptide gene enhancer in B-cells inhibitor, alpha	4792	5.2
NR4A1	nuclear receptor subfamily 4, group A, member 1	3164	5.4
IER3	immediate early response 3	8870	5.7
TNFAIP3	tumor necrosis factor, alpha-induced protein 3	7128	6.4
BTG2	BTG family, member 2	7832	7.0
TNFRSF9	tumor necrosis factor receptor superfamily, member 9	3604	8.2
BIRC3	baculoviral IAP repeat-containing 3	330	14.8
IL6	interleukin 6 (interferon, beta 2)	3569	18.0
	***Cell cycle regulation***		
GADD45A	growth arrest and DNA-damage-inducible, alpha	1647	2.2
RGS2	regulator of G-protein signaling 2, 24 kDa	5997	2.2
MAP3K8	mitogen-activated protein kinase kinase kinase 8	1326	2.3
SESN1	sestrin 1	27244	2.6
CDKN1A	cyclin-dependent kinase inhibitor 1A (p21, Cip1)	1026	2.8
CYLD	cylindromatosis (turban tumor syndrome)	1540	2.9
PLK2	polo-like kinase 2 (Drosophila)	10769	3.0
SMAD3	SMAD family member 3	4088	4.4
JUN	jun oncogene	3725	4.6
GADD45B	growth arrest and DNA-damage-inducible, beta	4616	4.8
DUSP1	dual specificity phosphatase 1	1843	8.5
IL8	interleukin 8	3576	41.7
	***Regulation of cell growth/Regulation of cell proliferation***		
ZMAT3	zinc finger, matrin type 3	64393	2.0
CSF1	colony stimulating factor 1 (macrophage)	1435	2.1
FGFR2	fibroblast growth factor receptor 2	2263	2.1
ABTB2	ankyrin repeat and BTB (POZ) domain containing 2	25841	2.4
SOCS2	suppressor of cytokine signaling 2	8835	2.4
PGF	placental growth factor	5228	2.6
HBEGF	heparin-binding EGF-like growth factor	1839	2.7
LIF	leukemia inhibitory factor (cholinergic differentiation factor)	3976	2.9
FGF18	fibroblast growth factor 18	8817	4.9
CCL2	chemokine (C-C motif) ligand 2	6347	6.7
IL11	interleukin 11	3589	7.4
RARRES1	retinoic acid receptor responder (tazarotene induced) 1	5918	7.5
IGFBP1	insulin-like growth factor binding protein 1	3484	32.2
DLGAP4	discs, large (Drosophila) homolog-associated protein 4	22839	2.5
EFNA1	ephrin-A1	1942	2.5
WNT4	wingless-type MMTV integration site family, member 4	54361	9.3
	***Cell adhesion***		
LYPD3	LY6/PLAUR domain containing 3	27076	2.0
PDZD2	PDZ domain containing 2	23037	2.1
FERMT2	fermitin family homolog 2 (Drosophila)	10979	2.2
NINJ1	ninjurin 1	4814	2.2
SIRPA	signal-regulatory protein alpha	140885	2.2
COL7A1	collagen, type VII, alpha 1	1294	2.3
LAMB3	laminin, beta 3	3914	2.6
CDH5	cadherin 5, type 2 (vascular endothelium)	1003	4.4
CTGF	connective tissue growth factor	1490	4.4
SAA1	serum amyloid A1///serum amyloid A2	6288	10.3
ICAM1	intercellular adhesion molecule 1	3383	10.8
	***Transport***		
SLC37A1	solute carrier family 37 (glycerol-3-phosphate transporter), member 1	54020	2.0
SLC1A3	solute carrier family 1 (glial high affinity glutamate transporter), member 3	6507	2.2
C19orf28	chromosome 19 open reading frame 28	126321	2.6
NPTX1	neuronal pentraxin I	4884	2.6
SLC2A6	solute carrier family 2 (facilitated glucose transporter), member 6	11182	3.5
HBA1	hemoglobin, alpha 1	3039	5.4
	***Metabolic process***		
HMGCS1	3-hydroxy-3-methylglutaryl-Coenzyme A synthase 1 (soluble)	3157	2.0
PTGS1	prostaglandin-endoperoxide synthase 1 (prostaglandin G/H synthase and cyclooxygenase)	5742	2.0
IDS	iduronate 2-sulfatase	3423	2.2
PI4K2A	phosphatidylinositol 4-kinase type 2 alpha	55361	2.2
PLA2G4C	phospholipase A2, group IVC (cytosolic, calcium-independent)	8605	2.2
C12orf5	chromosome 12 open reading frame 5	57103	2.3
PANX1	pannexin 1	24145	2.3
ABCA1	ATP-binding cassette, subfamily A (ABC1), member 1	19	2.4
AMPD3	adenosine monophosphate deaminase (isoform E)	272	2.4
SAT1	spermidine/spermine N1-acetyltransferase 1	6303	2.5
AKR1B1	aldo-keto reductase family 1, member B1 (aldose reductase)	231	2.6
GCNT3	glucosaminyl (N-acetyl) transferase 3, mucin type	9245	2.6
PITPNM1	phosphatidylinositol transfer protein, membrane-associated 1	9600	2.6
MICAL2	microtubule-associated monoxygenase, calponin and LIM domain containing 2	9645	3.2
PPAP2B	phosphatidic acid phosphatase type 2B	8613	3.2
ARG2	arginase, type II	384	4.1
PTGES	prostaglandin E synthase	9536	4.7
GFPT2	glutamine-fructose-6-phosphate transaminase 2	9945	5.0
	***Phosphorylation/Dephosphorylation***		
DUSP6	dual specificity phosphatase 6	1848	2.0
FAM129A	family with sequence similarity 129, member A	116496	2.3
DUSP13	dual specificity phosphatase 13	51207	2.6
DUSP5	dual specificity phosphatase 5	1847	4.1
PTPRE	protein tyrosine phosphatase, receptor type, E	5791	4.1
	***Response to stress***		
HSPA2	heat shock 70 kDa protein 2	3306	2.3
HSPA1A	heat shock 70 kDa protein 1A	3303	2.9
HSPB8	heat shock 22 kDa protein 8	26353	3.1
HSPB3	heat shock 27 kDa protein 3	8988	4.0
HSPB7	heat shock 27 kDa protein family, member 7 (cardiovascular)	27129	4.0
DNAJB1	DnaJ (Hsp40) homolog, subfamily B, member 1	3337	4.2
HSPA6	heat shock 70 kDa protein 6	3310	7.6
	***Ubiquitin***		
ENC1	ectodermal-neural cortex (with BTB-like domain)	8507	2.3
MAP1LC3C	microtubule-associated protein 1 light chain 3 gamma	440738	3.4
	***Others/Unknown***		
OLR1	oxidized low density lipoprotein (lectin-like) receptor 1	4973	2.0
TRIB1	tribbles homolog 1 (Drosophila)	10221	2.0
UNC13A	unc-13 homolog A (C. elegans)	23025	2.0
SNAI1	snail homolog 1 (Drosophila)	6615	2.1
FSTL3	follistatin-like 3 (secreted glycoprotein)	10272	2.2
GAB2	GRB2-associated binding protein 2	9846	2.2
PDLIM3	PDZ and LIM domain 3	27295	2.2
PMEPA1	prostate transmembrane protein, androgen induced 1	56937	2.2
SLC1A3	solute carrier family 1 (glial high affinity glutamate transporter), member 3	6507	2.2
FNDC3B	fibronectin type III domain containing 3B	64778	2.3
PHLDA3	pleckstrin homology-like domain, family A, member 3	23612	2.3
SLC25A4	solute carrier family 25 (mitochondrial carrier; adenine nucleotide translocator), member 4	291	2.3
TPM4	tropomyosin 4	7171	2.3
DSE	dermatan sulfate epimerase	29940	2.5
VEGFC	vascular endothelial growth factor C	7424	2.5
CSTA	cystatin A (stefin A)	1475	2.6
ZDHHC18	zinc finger, DHHC-type containing 18	84243	2.6
CDK2AP2	cyclin-dependent kinase 2 associated protein 2	10263	2.7
TIPARP	TCDD-inducible poly(ADP-ribose) polymerase	25976	2.7
CSTA	cystatin A (stefin A)	1475	2.8
TNFAIP2	tumor necrosis factor, alpha-induced protein 2	7127	2.8
PSD4	pleckstrin and Sec7 domain containing 4	23550	3.1
KRT17	keratin 17	3872	3.7
ARC	activity-regulated cytoskeleton-associated protein	23237	5.4
LXN	latexin	56925	5.5
TRIM31	tripartite motif-containing 31	11074	20.1

**Table 2:** Genes downregulated by Tax (fold change ≥ 2.0, p < 0.05)

**Gene symbol**	**Gene description**	**Gene ID**	**Fold change**
	***Transcription/Translation/RNA processing***		
ANP32A	Cerebellar leucine rich acidic nuclear protein (LANP)	8125	2.0
EID1	EP300 interacting inhibitor of differentiation 1	23741	2.0
PAIP1	poly(A) binding protein interacting protein 1	10605	2.0
RBM4	RNA binding motif protein 4	5936	2.0
SFRS7	splicing factor, arginine/serine-rich 7, 35 kDa	6432	2.0
SR140	U2-associated SR140 protein	23350	2.0
BCLAF1	BCL2-associated transcription factor 1	9774	2.1
IMPACT	Impact homolog (mouse)	55364	2.3
LSM5	LSM5 homolog, U6 small nuclear RNA associated (S. cerevisiae)	23658	2.3
MRPS14	mitochondrial ribosomal protein S14	63931	2.3
SUB1	SUB1 homolog (S. cerevisiae)	10923	2.3
ZNF623	zinc finger protein 623	9831	2.3
BRIP1	BRCA1 interacting protein C-terminal helicase 1	83990	2.4
TTF2	transcription termination factor, RNA polymerase II	8458	2.6
	***Signal transduction***		
PDE1A	phosphodiesterase 1A, calmodulin-dependent	5136	2.0
PDE3A	phosphodiesterase 3A, cGMP-inhibited	5139	2.0
PRKCI	protein kinase C, iota	5584	2.1
SRI	sorcin	6717	2.8
	***Immune response/Response to virus***		
DDX58	DEAD (Asp-Glu-Ala-Asp) box polypeptide 58	23586	2.2
IFI44	interferon-induced protein 44	10561	2.2
DDX60	DEAD (Asp-Glu-Ala-Asp) box polypeptide 60	55601	3.0
IFIT3	interferon-induced protein with tetratricopeptide repeats 3	3437	4.1
IFIT2	interferon-induced protein with tetratricopeptide repeats 2	3433	4.4
IFIT1	interferon-induced protein with tetratricopeptide repeats 1	3434	6.0
OASL	2'-5'-oligoadenylate synthetase-like	8638	6.4
	***Apoptosis***		
CARD10	caspase recruitment domain family, member 10	29775	2.0
BCLAF1	BCL2-associated transcription factor 1	9774	2.1
	***Cell cycle***		
MAD2L1	MAD2 mitotic arrest deficient-like 1 (yeast)	4085	2.0
KIF11	kinesin family member 11	3832	2.1
NF2	neurofibromin 2 (merlin)	4771	2.1
SEP11	septin 11	55752	2.1
CENPF	centromere protein F, 350/400 ka (mitosin)	1063	2.5
	***Regulation of cell proliferation***		
BMP2	bone morphogenetic protein 2	650	2.2
DAB2	disabled homolog 2, mitogen-responsive phosphoprotein (Drosophila)	1601	2.4
FGF2	fibroblast growth factor 2 (basic)	2247	3.4
	***Cell signaling***		
PCSK1	proprotein convertase subtilisin/kexin type 1	5122	4.8
	***Cell adhesion***		
CD24	CD24 molecule	100133941	2.3
PKP2	plakophilin 2	5318	2.3
COL14A1	collagen, type XIV, alpha 1	7373	2.5
	***Nucleosome assembly***		
H2AFV	H2A histone family, member V	94239	2.0
	***Transport***		
CNGB1	cyclic nucleotide gated channel beta 1	1258	2.0
SCNN1A	sodium channel, nonvoltage-gated 1 alpha	6337	2.0
ANO2	anoctamin 2	57101	2.1
CHRNA9	cholinergic receptor, nicotinic, alpha 9	55584	2.2
SORBS1	sorbin and SH3 domain containing 1	10580	2.3
STEAP4	STEAP family member 4	79689	2.3
SRI	sorcin	6717	2.6
	***Metabolic process***		
ACSL4	acyl-CoA synthetase long-chain family member 4	2182	2.0
	***Ubiquitin***		
FBXO3	F-box protein 3	26273	2.0
HERC6	hect domain and RLD 6	55008	2.1
DZIP3	DAZ interacting protein 3, zinc finger	9666	2.3
HERC5	hect domain and RLD 5	51191	4.8
	***Others/Unknown***		
NIP7	nuclear import 7 homolog (S. cerevisiae)	51388	2.0
PPL	periplakin	5493	2.0
ADK	adenosine kinase	132	2.1
DIO2	deiodinase, iodothyronine, type II	1734	2.1
PICALM	phosphatidylinositol binding clathrin assembly protein	8301	2.1
METAP2	methionyl aminopeptidase 2	10988	2.2
HIP1R	huntingtin interacting protein 1 related	9026	2.3
ERAP1	KIAA0525 protein	51752	2.4
DAB2	disabled homolog 2, mitogen-responsive phosphoprotein (Drosophila)	1601	2.5

The upregulated genes (2-fold or greater) were clustered within functional groups involved in transcription/translation/RNA processing, signal transduction, the immune response, apoptosis, cell cycle regulation, and cell growth/proliferation (Table 1). In addition, a number of molecules involved in the immune response were significantly downregulated by Tax (Table 2).

### Tax induces the expression of genes related to cell cycle progression and apoptosis

**Figure 2:**
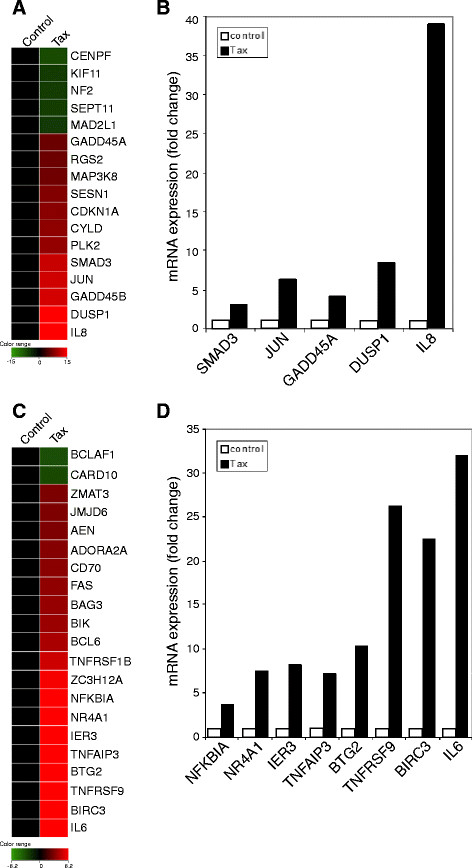
**Expression profile of genes involved in cell cycle regulation and apoptosis that were altered following the induction of Tax protein.** Heat maps showing the hierarchical clustering of genes involved in cell cycle regulation (**A**) and apoptosis (**C**) are shown for Tax-expressing cells. The color scheme indicates the fold change in gene expression, with upregulated genes shown in orange/red and downregulated genes shown in blue (with respect to baseline levels under control conditions (yellow)). qRT-PCR validation of the upregulated genes associated with cell cycle regulation (**B**) and apoptosis (**D**). RNA from Tax-expressing cells and control cells was used to validate the microarray data. The bars indicate the fold change in gene expression following Tax expression. Data were normalized to GAPDH mRNA. The results represent the mean of two samples from one experiment

It was hypothesized that changes in gene expression may provide valuable information about the dysregulation of cell cycle progression induced by Tax and about how Tax might affect the genes relevant to this process. As shown in Figure 2A, of 17 genes related to cell cycle progression that were regulated by Tax, five were downregulated and 12 were upregulated (fold change > 2.0; p < 0.05). Genes associated with mitosis (CENPF, SEP11, and NF2), including the mitotic cell cycle checkpoint (MAD2L1) and mitotic centrosome separation (KIF11), were repressed by Tax. By contrast, genes upregulated by Tax were functionally classified as genes related to the cell cycle (GADD45A, RGS2, MAP3K8, SESN1, CDKN1A, CYLD, PLK2, SMAD3, JUN, GADD45B, DUSP1 and IL8). Many of these genes are also involved in other processes, such as the response to stress (GADD45B), the response to DNA damage (GADD45A, SESN1, CDKN1A), MAP kinase activity (GADD45B, MAP3K8, DUSP1), cell proliferation (JUN and IL8), and negative regulation of the cell cycle (CYLD, PLK2 and SMAD3). Genes such as SMAD3, GADD45B, and DUSP1 were also identified as having a role in apoptosis, and IL8 is additionally involved in inflammation and the immune response.

The microarray results for genes related to cell cycle progression were validated by performing real-time quantitative reverse transcription polymerase chain reaction (qRT-PCR) on five upregulated genes (Figure 2B). The results of the qRT-PCR agreed with those obtained by microarray analysis.

Next, Tax-regulated genes related to apoptosis were identified (Figure 2C). The microarray results revealed that 21 pro- or anti-apoptotic genes were regulated by Tax (fold change > 2.0; p < 0.05). Two genes associated with the induction of apoptosis, CARD10 and BCLAF1, were downregulated by Tax. The majority of the genes upregulated by Tax were involved in apoptosis. Furthermore, several of these genes also function in the immune response (ADORA2A, CD70, FAS, BCL6, TNFRSF1B and IL6). Interestingly, several highly upregulated genes, such as IER3, TNFAIP3, BIRC3 and IL6, have both pro- and anti-apoptotic functions. In contrast, the highly upregulated gene, TNFRSF9, is pro-apoptotic only. TNF and TNF receptor family genes were also found to be upregulated by Tax in this study.

To confirm and extend the results of the microarray experiments, expression of the pro-apoptotic and anti-apoptotic genes regulated by Tax was measured by qRT-PCR using specific primers. Genes upregulated in the microarray were also upregulated in qRT-PCR (Figure 2D), although there were small differences in the levels measured by the two methods. For example, the expression levels of BIRC3 and IL6 measured by qRT-PCR were almost twice that measured by microarray analysis, and the expression level of the apoptosis inductor TNFRSF9 was more than three times higher by qRT-PCR than by microarray. Despite these minor differences, overall gene expression levels measured by qRT-PCR were similar to those measured by microarray analysis.

### Visualizing the spatiotemporal dynamics of the regulation of cell cycle progression and apoptosis by tax

**Figure 3:**
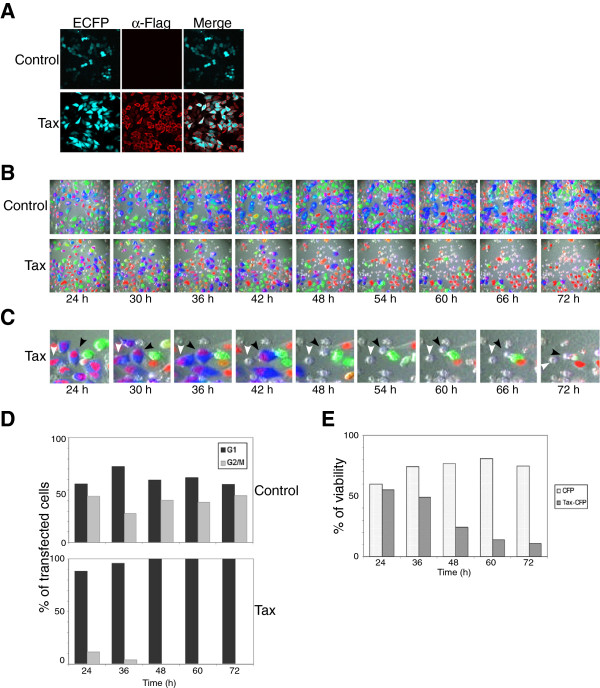
**Time-lapse imaging of morphological changes in HeLa/Fucci2 cells after Tax-induced cell cycle arrest at G**_**1**_**phase.** (**A**) HeLa cells were transfected with the pCAGGS-Tax-IRES-CFP vector or the control pCAGGS-IRES-CFP vector. At 24 h after transfection, cells were stained with an anti-Flag MAb followed by an Alexa Fluor 594-conjugated secondary MAb and analyzed by confocal laser scanning microscopy (Olympus FV1000). Cells showing red and blue fluorescence express Tax-Flag and CFP, respectively. (**B** and **C**) HeLa/Fucci2 cells were transfected with the CAGGS-Tax-IRES-CFP vector or the control pCAGGS-IRES-CFP vector and monitored by time-lapse photography using the Olympus LCV110 Imaging System. One day after transfection, CFP-positive cells were selected and fluorescence and phase images were captured once every 15 min for 2 days. Cells showing orange or green fluorescence are in the G_1_ or S/G_2_/M phase of cell cycle, respectively. Apoptotic cells, which show a rounded morphology, are marked by arrows. The populations of CFP-expressing cells at the G_1_ and S/G_2_/M phases (**D** and **E**, respectively) were quantified using MetaMorph 7.7.4 software

To clarify whether Tax causes apoptosis independently of its ability to induce G_1_ arrest, the spatiotemporal patterns of cell cycle regulation in response to Tax expression were monitored in HeLa/Fucci2 cells [33]. This system was chosen because it allows dual-color imaging, in which G_1_-phase nuclei are labeled orange and S/G_2_/M-phase nuclei are labeled green. A fluorescent Tax vector was constructed that allows the identification of Tax-expressing HeLa/Fucci2 cells. This vector contained Tax, an internal ribosomal entry site (IRES), cyan fluorescent protein (CFP), and a Flag sequence at the 3’ end of *tax*. The vector was expressed in HeLa cells, and Tax-expressing cells were stained with an anti-Flag MAb followed by an Alexa Fluor 594 secondary antibody (red). As shown in Figure 3A, all Tax-expressing cells were CFP-positive (blue).

HeLa/Fucci2 cells were plated on a glass coverslip, transiently transfected with Tax-IRES-CFP or the CFP control vector, and then incubated for 24 h. Next, fields containing orange, green, and blue fluorescence were selected and images were acquired using an Olympus LCV110 Imaging System (Figure 3B and 3C). The proliferation of control HeLa/Fucci2 cells was evidenced by the fraction of cells at G_1_ phase with orange nuclei, the fraction of cells at S/G_2_/M phase with green nuclei, and the subsequent change in the fluorescence of these cells (Figure 3B upper panel and 3D), which indicated that the cells progressed normally through the cell cycle. At 24 h post-transfection, all HeLa/Fucci2 cells expressing Tax-IRES-CFP, which resulted in blue fluorescence, also had orange nuclei, indicating that they were in G_1_ phase (Figure 3B, lower panel). During the culture period, HeLa/Fucci2 cells expressing Tax-IRES-CFP did not progress to S/G_2_/M phase, as evidenced by the presence of orange nuclei and the absence of green nuclei in Tax-expressing cells (Figure 3B). Additionally, a marked decrease was observed in the proportion of Tax-IRES-CFP-expressing cells in S/G_2_/M phase compared with control cells expressing CFP alone (Figure 3D), indicating that Tax arrests cells at the G_1_ phase of the cell cycle.

Interestingly, overexpression of Tax appeared to reduce the number of HeLa/Fucci2 cells in culture (Figure 3E). Moreover, apoptosis was assessed by the appearance of rounded cells after an increase in the number of Tax-expressing cells at G_1_ phase, starting at 36 h post-transfection (Figure 3B and 3C). At 72 h post transfection, there was a notable reduction in the overall number of cells, as well as in the percentage of Tax-expressing cells (Figure 3C and 3E).

### Expression kinetics of genes involved in cell cycle regulation and apoptosis that are altered following induction of tax protein

**Figure 4:**
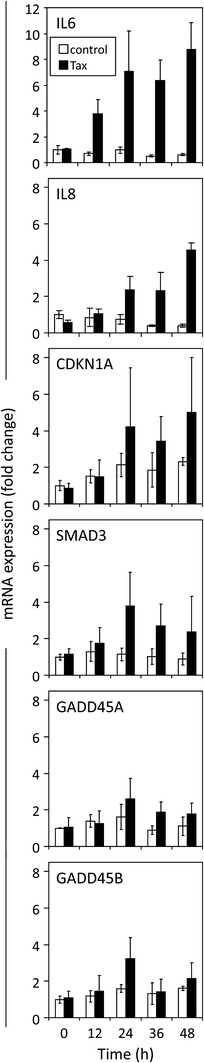
**Expression kinetics of genes involved in cell cycle regulation and apoptosis that were altered following induction of Tax.** HeLa cells were transiently transfected with a pCAGGS-Tax Flag-tagged vector or the control pCAGGS vector. Total RNA was prepared at 12, 24, 36 and 48 h after transfection and then each RNA sample was subjected to qRT-PCR. The bars indicate the fold change in the gene following Tax expression. Data were normalized to GAPDH mRNA. The results represent the mean ± standard deviation (SD) of three samples from one experiment

To analyze the correlation between the expression of genes related to cell cycle regulation (IL8, SMAD3, CDKN1A, GADD45A and GADD45B) and apoptosis (IL6) (that are altered following the induction of Tax) with the dynamics of cell cycle and apoptosis (shown in Figure 3), total RNA was prepared at 12, 24, 36 and 48 h after transfection of HeLa cells with Tax or a control vector. Each RNA sample was then subjected to qRT-PCR. As indicated in Figure 4, the expression levels of SMAD3, GADD45A and GADD45B in Tax-transfected cells began to increase from 6 h post-transfection and reached a peak at 24 h, decreasing again by 36 h. In the case of IL8, CDKN1A and IL6 in Tax-expressing cells, the expression levels reached a peak at 24 h, decreased at 36 h, and then increased again at 48 h.

The kinetics and results from time-lapse imaging indicate that marked upregulation of IL8, SMAD3, CDKN1A, GADD45A, GADD45B and IL6 at 24 h post-transfection was well correlated with a notable reduction in the number of Tax-expressing cells and an increase of Tax-expressing cells in the G_1_ phase.

## Discussion

This study used large-scale host cell gene profiling with human cDNA microarrays and time-lapse imaging of HeLa/Fucci2 cells to monitor the dynamics of Tax-induced cell death. Three major conclusions can be drawn from the data: (i) Tax induces cell cycle arrest at the G_1_ phase in HeLa cells as assessed by flow cytometry. This result was confirmed by the accumulation of hypo- and/or unphosphorylated form of Rb in Tax-expressing cells. Moreover, analysis of Annexin V-stained cells and caspase-3 activity clearly demonstrated that Tax promotes apoptosis. Thus, a high level of transiently-expressed Tax can arrest the cell cycle at the G_1_ phase and induce apoptosis in HeLa cells. (ii) The most interesting aspect of this study was visualizing the morphological dynamics of Tax-induced cell death after cell cycle arrest at the G_1_ phase. Time-lapse imaging of HeLa/Fucci2 cells showed that Tax-induced apoptosis was dependent on the ability of Tax to induce G_1_ arrest. (iii) Microarray data revealed that Tax induced gene expression changes in HeLa cells; 17 Tax-dependent genes were found to be related to cell cycle regulation and 23 to apoptosis (> 2.0-fold up- or downregulation). (iv) The kinetics of gene expression identified that Tax-induced changes in the expression of IL8, SMAD3, CDKN1A, GADD45A, GADD45B and IL6 closely correlated with the morphological changes of the cell cycle and apoptosis observed by time-lapse imaging. Since these genes are related not only to cell cycle regulation and apoptosis induction, but also to stress kinase pathways, the present study suggests that Tax may induce apoptosis and cell cycle arrest by activating genes related to stress-response signaling pathways.

Many studies show that the Tax oncoprotein accelerates G1 progression [3,4,7-12] and is capable of stimulating anti-apoptotic signaling pathways [29,30,36,37]. In contrast, the present study showed that Tax arrests cells at G_1_, thereby inducing apoptosis. Our results consist with previous results obtained using HeLa cells and SupT1 cells [20,38]. There may be possible explanations for how Tax induces cell cycle arrest and apoptosis. One interesting finding from our microarray analysis was the marked activation of stress kinase pathways induced by Tax. In mammalian cells, two families of stress-responsive MAPKs, c-Jun N-terminal kinase (JNK) and p38, are activated by stimuli such as UV radiation, oxidative stress and translation inhibitors, as well as by inflammatory cytokines, tumor necrosis factor α (TNFα), and transforming growth factor β (TGFβ). These signaling pathways promote apoptosis, cell survival, cell cycle arrest, inflammation and differentiation [39,40]. Interestingly, microarray analysis revealed that genes such as SMAD3 and SMAD4, which are the principal intracellular effectors of the TGFβ family [41,42]; GADD45A and GADD45B, which are implicated as stress sensors and activated by TGFβ in a SMAD-dependent manner [43-45]; DUSP1, DUSP5, DUSP6 and DUSP13, which are stress-inducible MAP kinase phosphatases [46]; MAP kinase kinase kinase 8 (MAP3K8) [46]; JUN [46], which is the effector transcription factor of the JNK pathway; and IL6, IL8 and FAS, which are inflammatory cytokines, were all upregulated by Tax. These genes, expressed in response to Tax, are mediators of JNK and p38 activity. In addition, we found that the kinetics of altered expression of several genes related to pathways involving stress-responsive MAPKs were closely correlated with the kinetics of the spatial and temporal patterns of cell cycle dynamics analyzed in time-lapse imaging. At 24 h post-transfection with Tax expression vectors, the genes for IL8, SMAD3, CDKN1A, GADD45A, GADD45B and IL6 were significantly upregulated (Figure 4) and the number of Tax-IRES-CFP-expressing cells were in G_1_ phase and underwent apoptosis started to increase at same timing (Figure 3). Thus, the present results suggest that Tax may induce apoptosis and cell cycle arrest by activating several genes related to stress-response signaling pathways. This is supported by a recent publication showing that Tax, along with the activation of a stress kinase, can induce cell death [31]. Furthermore, the present findings consist with those observed by previous microarray analysis studies of HTLV-1-infected T cells, which demonstrated that HTLV-1 infection upregulated JNK activation kinase 1, GADD45 and the inflammatory cytokine, IL1β, which are involved in MAPK stress-response pathways [23]. Recently, HTLV-1 Tax appeared indirectly to connect to cell cycle proteins such as SMAD3, SMAD4, GADD45A and GADD45B [47].

Our microarray analysis results identified one of the genes upregulated by Tax as *CDKN1A*, which codes p21^CIP1/WAF1^, known as Cdk inhibitor 1. Again, this is in agreement with results from other microarray analyses showing that HTLV-1 infection and Tax expression upregulated p21^CIP1/WAF1^ in HTLV-1-infected T cells [23] and the human Jurkat T-cell line JPX-9, which express Tax under the control of an inducible promoter [48]. Likewise, Tax has previously been shown to dramatically upregulate p21^CIP1/WAF1^ mRNA transcription and stabilization of p21^CIP1/WAF1^ in HeLa cells [20,21]. Interestingly, only minimal p21/WAF1 promoter activity appears to be induced by Tax [23]. It is also known that basal levels of p21^CIP1/WAF1^ are required to promote TGFβ-mediated cell cycle arrest, whereas a lack of p21^CIP1/WAF1^ allows the induction of cell proliferation in response to TGFβ [49]. Indeed, the loss of p21^CIP1/WAF1^ and p27^KIP1^ from HOS cells apparently allows HTLV-1-and Tax-induced G1 arrest to be bypassed [20]. Therefore, Tax may induce cell cycle arrest and apoptosis in HeLa cells by up-regulating GADD45B, SMAD3 and SMAD4 (which act downstream of TGFβ) in the presence of p21^CIP1/WAF1^ (which is activated by Tax).

In HTLV-1 infected T cell lines, upregulated p21^CIP1/WAF1^ may potentially function as an assembly factor for the cyclin D2/cdk4 complex, and the p21/cyclin D2/cdk4 complex may not act as an inhibitory complex but instead may allow the increased phosphorylation of Rb and accelerated progression into S phase [50]. In the present study, Tax-mediated G1 arrest occurred in human papilloma virus type 18 (HPV-18)-transformed HeLa cells, in which the Rb pathway was activated by repression of HPV-18 E7 [51]. Indeed, in cells transfected with the control vector, the majority of Rb was in the hyperphosphorylated form ppRb (Figure 1E). By contrast, an accumulation of hypo- and/or unphosphorylated form pRb was observed in Tax-expressing HeLa cells, which is in contrast to the results of study showing that Tax increased the phosphorylation of Rb family members [19]. Therefore, there is a strong possibility that Tax-activated p21^CIP1/WAF1^ may function to inhibit the cyclin D2/cdk4 complex, thereby inducing cell cycle arrest.

Our microarray result also shows that Tax upregulated the expression of BCL6 gene encodes a sequences-specific transcriptional repressor by 2.7 fold. This supported by the findings in previous study [52], which described that an interaction of Tax with the POZ domain of BCL6 enhances the repressive activity of BCL6 and increased the levels of apoptosis induced by BCL6 in osteosarcoma cells. The BCL6 POZ domain mediates transcriptional repression by interacting with several corepressors including silencing mediator for retinoid and thyroid receptor and nuclear hormone receptor corepressor, BCL6 corepressor together with many histone deacetylases. BCL6 colocalizes with these corepressors in punctate nuclear structures that have been identified as sites of ongoing DNA replication. Interestingly, BCL6 appeared to recruite Tax into punctate nuclear structures and significantly downregulate both basal and Tax-induced NF-kB and long terminal repeat activation [52]. Thus, the high expression of BCL6 in HTLV infected cells may contribute to the silencing of viral gene expression and to the long clinical latency associated with HTLV infection.

This study allows greater understanding of the biological events affected by HTLV-1 Tax, particularly the regulation of cellular proliferation and apoptosis. Since we found evidence of several similarities, as well as differences, between Tax-expressing HeLa cells and HTLV infection in T cell lines, we believe that the overexpression of Tax will be useful for preliminary studies on the effects of HTLV infection in T cell lines. However, since Zane *et al.* recently demonstrated that infected CD4^+^ T cells *in vivo* are positively selected for cell cycling but not cell death [53], our experimental approaches in HeLa cells may not be reflective of normal physiology of Tax or HTLV-1 *in vivo* infected cells. Therefore, further detailed studies are required to define the direct and indirect effects of Tax-mediated cellular processes to gain a better understanding of the contribution of Tax to HTLV-1 pathogenesis *in vivo*.

## Conclusion

The present study showed that Tax arrested cells at the G_1_ phase of the cell cycle, thereby inducing apoptosis. Taken together, the results demonstrate that Tax exerts a significant impact on cellular factors that regulate the cell cycle and the induction of apoptosis. Importantly, to the best of our knowledge, this is the first study to highlight the morphological dynamics of Tax-induced cell death after cell cycle arrest at the G_1_ phase.

This overview can be extended to Tax-mediated signaling, and further study of the interactions between Tax and cellular factors will provide insights into the mechanisms by which Tax regulates host cell behavior, as well as the mechanisms underlying lymphoma induction and progression induced by HTLV-1.

## Methods

### Cell lines and transfections

Human cervical HeLa cells and Fucci2-expressing HeLa cells (HeLa/Fucci2) [33] were maintained in Dulbecco’s modified Eagle’s medium (DMEM) (Invitrogen) supplemented with 10% heat-inactivated fetal bovine serum (FBS) and 100 units/ml penicillin/streptomycin (Sigma). Cells were transiently transfected with a Tax expression vector, or a control vector, using Fugene HD (Roche) according to the manufacturer’s instructions.

### Plasmid construction

The HTLV-1 *tax* gene was amplified from the HTLV-1 infectious molecular clone, K30 [54], using the primers HTax-F (5’-3’, AACTCGAGGCCACCATGGCCCATTTCCCAGGGTTTGGAC) and HTax-R (5’-3’, AAGCGGCCGCTCACTTGTCGTCATCGTCTTTGTAGTCGACTTCTGTTTCTCGGAAATGTTTTTCACTGG). The underlined sequences correspond to restriction enzyme sites specific for *Xho*I and *Not*I, respectively. A Flag sequence was included at the 3’ end of the *tax* gene. Full-length *tax* was then cloned into the *Xho*I and *Not*I restriction sites in the pCAGGS mammalian expression vector [55]. To generate the pCAGGS-Tax-IRES-CFP vector and the pCAGGS-IRES-CFP control vector, the IRES was amplified from the pRetroX-IRES-ZsGreen1 vector (Clontech) and CFP was amplified from the pCS2+ vector (Clontech). The IRES and CFP sequences were then inserted into the pCAGGS control vector or a pCAGGS vector containing Flag-tagged Tax. The vector pEGFP-N1 encodes a red-shifted variant of wild-type GFP that was modified for brighter fluorescence [56] and which was used as a reporter to identify transfected cells by flow cytometry. The pSV-β-galactosidase vector (Promega) encoding a bacterial β-galactosidase and pRL-SV40 (Promega) encoding *Renilla* luciferase were used to normalize the transfection efficiency. pGV-HL21 encodes five tandemly repeated 21 bp enhancers of HTLV-1, each of which contain a CRE motif and pGV(−) and have been previously decribed [57].

### RNA extraction

HeLa cells were transiently transfected with Tax or the control vector and incubated for 30 h. RNA from total cell extracts was isolated using the RNeasy Mini Kit (Qiagen) according to the manufacturer's instructions. RNA was quantified using a spectrophotometer and stored at −80°C. For gene chip analysis, the quality of RNA was determined using the Agilent Bioanalyzer (Agilent Technologies).

### Microarray analysis

RNA samples were analyzed by microarray using the GeneChip Human Genome U133A 2.0 Array (Affymetrix). Microarray hybridization and fluorescence detection were performed as described in the Affymetrix Gene-Chip Expression Analysis Technical Manual. Microarray data were deposited in NCBI’s Gene Expression Omnibus and assigned GEO Series accession number GSE34750. GeneSpring GX 11.0 software (Agilent Technologies) was used to identify statistically significant differences in gene expression between samples. For multiple measurements to detect significantly upregulated and downregulated genes, the Bonferroni correction was performed by adjusting the significance level (p < 0.05). Fold changes in gene expression, hierarchical clustering, and gene ontology annotations were determined.

### qRT-PCR

Total RNA was prepared using the RNeasy Mini Kit (Qiagen) at 12, 24, 36 and 48 h after transfection with Tax or the control vector. RT-PCR was performed using specific primers and OneStep SYBR Green PCR mix (Takara) following the manufacturer’s instructions. The qRT-PCR was performed using a 7500 Fast Real-time PCR System (Applied Biosystems). All data were normalized to GAPDH mRNA.

### Immunoblot analysis

Transfected cells were lysed and proteins were separated on 6%, 10%, or 17% SDS-polyacrylamide gels and then transferred to a PVDF membrane (Immobilon-P, Millipore Corp.) using a Trans-blot SD semi-dry transfer cell (Bio-Rad). Following the transfer, the membranes were blocked in 5% non-fat dry milk in PBS containing 0.1% Tween-20 for 1 h and then incubated with a 1:1000 dilution of primary antibody against Flag (M2, Sigma), Rb (c-15, Santa Cruz Biotechnology), or actin (c-11, Santa Cruz Biotechnology) for 1 h. The membranes were then washed and incubated with anti-mouse, anti-rabbit, or anti-goat horseradish peroxidase-conjugated secondary antibodies (Jackson, ImmunoResearch) and developed using the SuperSignal West Pico Chemiluminescent substrate Kit (Pierce).

### Immunofluorescence

Cells (1 x 10^5^) were seeded onto 22 mm diameter coverslips in 24-well plates and incubated at 37°C for 24 h before transfection. Cells were transiently transfected with either a Tax expression vector or a control vector using the Fugene HD reagent (Roche). Twenty-four hours later, the cells were washed twice with PBS, fixed in 3.7% formaldehyde, permeabilized using 0.2% Triton X-100, and stained with an anti-Flag MAb (M2, Sigma) followed by an anti-mouse IgG1 antibody conjugated to Alexa Fluor 488 or 494 (Molecular Probes). Subcellular localization was analyzed by confocal laser scanning microscopy (FV1000, Olympus).

### Luciferase assay

HeLa cells (1 x 10^5^) were transfected with 1 μg of the reporter plasmid, pGV-HL21 (HTLV-1 enhancer) or pGV(−), 0.3 μg of the reference plasmid, pRL-SV40, and 0.5 μg of the Tax expression vector. At 48 h after transfection, cells were recovered and the activity of firefly and *Renilla* luciferase was measured in the lysates as previously described [58]. For each sample, firefly luciferase activity (pGV-HL21) was normalized by reference to *Renilla* luciferase activity (pRL-SV40).

### Cell cycle analysis

HeLa cells (4 x 10^5^) were incubated in a 6-well plate at 37°C for 24 h followed by co-transfection for 48 h with 2 μg of the Tax expression vector or the control vector and 0.2 μg of the pEGFP-N1 vector. Cells were collected and washed with PBS without Ca^2+^ and Mg^2+^ and then fixed with 1% paraformaldehyde followed by 70% ethanol. After fixation, cells were washed twice with PBS, treated with 200 μg/ml of RNase for 1 h at 37°C, and stained with 50 μg/ml of PI. Fluorescence was analyzed using a FACSCalibur (Becton-Dickinson) flow cytometer and Cell Quest software (Becton-Dickinson). Samples were gated to eliminate cells in which GFP emitted strong fluorescence. The acquired FACS data were analyzed using ModFit LT software (Verity Software House).

### Analysis of apoptosis

Flow cytometry was used to detect Annexin V-positive apoptotic cells. Transfected cells were incubated for 48 h and then the cell monolayers were detached with trypsin and ethylendiaminetetraacetic acid (EDTA), washed twice in PBS, and re-suspended in binding buffer (1 x 10^6^ cells/ml). An aliquot of 1 x 10^5^ cells was stained with 7-AAD and Annexin V-PE (BD Biosciences) for 15 min at room temperature according to the manufacturer's instructions and then analyzed on a FACSCalibur flow cytometer (BD Biosciences) with Cell Quest software (BD Biosciences). Cells were considered to be in the early stages of apoptosis if they showed staining for Annexin V-PE but not 7-AAD. The double-positive population was considered to be in the late stages of apoptosis, or already dead.

Caspase-3 activity was measured using a caspase-3/CPP32 fluorometric assay kit, according to the manufacturer's instructions. Briefly, transfected HeLa cells were harvested, washed twice with PBS, and treated with lysis buffer. Cell lysates were centrifuged at 15000 × g for 10 min at 4°C, supernatants were collected, and protein concentrations were determined with the Pierce BCA protein assay kit (Thermo Scientific). For each experimental point, 50 μg of total protein extract was incubated with the substrate for 2 h at 37°C. Caspase activity was quantified spectrophotometrically at a wavelength of 405 nm using a multi-label counter (Model 1420, Wallac Arvo, Perkin Elmer Life Sciences).

### Imaging of cultured cells

HeLa/Fucci2 cells were transiently transfected with Tax-IRES-CFP or the control vector and were subjected to long-term, time-lapse imaging using a computer-assisted fluorescence microscope (Olympus, LCV110) equipped with an objective lens (Olympus, UAPO 40×/340 N.A. = 0.90), a halogen lamp, a red LED (620 nm), a CCD camera (Olympus, DP30), differential interference contrast (DIC) optical components, and interference filters. For fluorescence imaging, the halogen lamp was used with three filter cubes for observing mCherry (orange), Venus (green), and CFP (blue) fluorescence. For DIC imaging, the red LED was used with a filter cube containing an analyzer. Image acquisition and analysis were performed using MetaMorph 7.7.4 software (Universal Imaging).

## Competing interests

The authors declare that they have no competing interests.

## Authors' contributions

MA performed the experiments, analyzed the data, and wrote the manuscript. HM performed the qRT-PCR and analyzed the data. YA conceived the study, participated in the experimental design, analyzed and interpreted the results, coordinated experiments, and wrote the manuscript. All authors have read and approved the final manuscript.
